# Examining the relationship between rheumatoid arthritis, multimorbidity and adverse health-related outcomes: A systematic review protocol

**DOI:** 10.1177/2235042X20906657

**Published:** 2020-03-16

**Authors:** Jordan Canning, Stefan Siebert, Bhautesh D Jani, Frances S Mair, Barbara I Nicholl

**Affiliations:** 1 General Practice and Primary Care, Institute of Health and Wellbeing, College of Medical, Veterinary and Life Sciences, University of Glasgow, Glasgow, UK; 2 Institute of Infection, Immunity and Inflammation, College of Medical, Veterinary and Life Sciences, University of Glasgow, Glasgow, UK

**Keywords:** Arthritis, rheumatoid, comorbidity, multimorbidity, mortality, outcome assessment (healthcare)

## Abstract

**Background:**

Rheumatoid arthritis (RA) is a chronic autoimmune disorder characterised by articular inflammation and systemic complications. Multimorbidity (the presence of two or more long-term health conditions) is highly prevalent in people with RA but the effect of multimorbidity on mortality and other health-related outcomes is poorly understood.

**Objective:**

To determine what is known about the effect, if any, of multimorbidity on mortality and health-related outcomes in individuals with RA.

**Design:**

Systematic review of the literature. The following electronic medical databases will be searched: MEDLINE, Embase, Cumulative Index to Nursing and Allied Health Literature, PsycINFO, The Cochrane Library and Scopus. Included studies will be quality appraised using the Quality in Prognostic Studies tool developed by the Cochrane Prognosis Methods Group. A narrative synthesis of findings will be undertaken and meta-analyses considered, if appropriate. This protocol adheres to the Preferred Reporting Items for Systematic Reviews and Meta-Analyses Protocols 2015 guidelines, ensuring the quality of the review.

**Conclusions:**

Understanding the influence of multimorbidity on mortality and other health-related outcomes in RA will provide an important basis of knowledge with the potential to improve future clinical management of RA. PROSPERO registration number: CRD42019137756.

## Introduction

### Rationale

Rheumatoid arthritis (RA) is the most prevalent inflammatory arthropathy, affecting 0.5–1.0% of the adult population in developed countries.^1^ In RA, persistent autoimmune-mediated inflammation causes pain, swelling, tenderness and destruction of synovial joints which is associated with functional disability and reduced quality of life.^2^ High economic costs and increased morbidity and mortality are often a consequence of RA and contribute substantially to the overall burden of disease at both patient and healthcare level.^3^ The management of RA has been transformed in recent decades by the optimal use of disease-modifying anti-rheumatic drugs and the availability of targeted biological treatments.^4^ However, the presence of concurrent long-term conditions (LTCs) can cause additional complications in individuals with RA.^5^ Common comorbid LTCs include cardiovascular disease, osteoporosis and depression, with the average RA patient having 1.6 additional LTCs; this number increases with age, disease duration and disease activity. It is estimated that multimorbidity (the co-existence of two or more LTCs) is present in approximately two-thirds of people with RA.^6^ Multimorbidity itself has been linked to excess mortality, increased healthcare utilisation and poorer quality of life.^7,8^ However, the relationship between RA and multimorbidity is poorly understood. Likewise, the effects of this relationship on mortality and other health-related outcomes remain unclear. Examining such relationships will provide a valuable foundation to support future patient care through greater understanding of those at increased risk of adverse health-related outcomes.

### Objectives

This systematic review will appraise the literature to determine what is known about the effect, if any, of multimorbidity on mortality and other health-related outcomes in individuals with RA.

## Methods

The Preferred Reporting Items for Systematic Review and Meta-Analysis Protocols (PRISMA-P) 2015 reporting guidelines were used to prepare this protocol (Online Supplementary File S1).^9,10^

### Eligibility criteria

Inclusion and exclusion criteria are summarised in Table 1.

**Table 1.** Summary of inclusion and exclusion criteria to be used during the study selection process.

Category	Inclusion criteria	Exclusion criteria
Study design	Longitudinal cohort studies (retrospective and prospective)	Cross-sectional studies; intervention studies; qualitative studies; case reports/series; review articles (including systematic reviews)
Population	Adults (18 years or older)	Children/adolescents (under 18 years); animal studies
Exposure	RA + ≥2 LTCs	RA + 1 LTC
Comparator	RA + ≤1 LTC	None
Outcomes	Mortality and/or health-related outcomes	
Publication type	Full-text published article	Conference abstracts; dissertations/theses; editorials/commentaries/letters
Language	English language	Non-English language

RA: rheumatoid arthritis; LTC: long-term condition.

#### Study design

Empirical studies using quantitative methods will be eligible for our review. We will expressly target longitudinal cohort studies (both retrospective and prospective). Cross-sectional studies, intervention studies, qualitative studies, case reports/series and review articles (including systematic reviews) will be excluded. There will be no restriction on the publication date of included studies in the systematic review, with a search end date of 6 December 2018.

#### Population

Studies must involve adults (18 years or older) with RA and two or more other physical or mental health LTCs. Studies involving children/adolescents (under 18 years) or animals will be excluded.

#### Exposure

The exposure of interest is the presence of two or more LTCs in adults with RA. We will include studies that assess the relationship between RA, multimorbidity and our outcomes of interest using any numerical count of comorbidity/multimorbidity, where the type of comorbid conditions is also specified. Studies that focus solely on one comorbid condition with RA will be excluded as this systematic review is only interested in the relationship between RA and multimorbidity.

#### Comparators

Participants with only RA and no other LTCs or those with RA and one other LTC will act as the comparator/control group. However, studies that do not include a comparator/control group will not be excluded.

#### Outcomes

The primary outcome will be all-cause mortality. Health-related outcomes will also be of interest, particularly those relating to disability and quality of life. Of interest, are those reported by patients regarding their emotional, social and physical wellbeing via health assessment questionnaires (HAQ) and/or patient-reported outcome measures. Studies which provide data on any of these outcomes will be included.

#### Publication type

Studies must be full-text published articles. Conference abstracts, dissertations/theses and editorials/commentaries/letters will be excluded.

#### Language

Studies must be in the English language.

### Information sources

The following electronic medical databases will be searched: MEDLINE (Ovid), Embase (Ovid), Cumulative Index to Nursing and Allied Health Literature (CINAHL; EBSCOhost), PsycINFO (EBSCOhost), The Cochrane Library (Central Register of Controlled Trials (CENTRAL); Wiley) and Scopus (Elsevier). The International Prospective Register of Systematic Reviews (PROSPERO) will be monitored for other ongoing and completed systematic reviews on RA and multimorbidity.

### Search strategy

The search strategy will combine the following concepts: (1) RA, (2) comorbidity/multimorbidity, (3) mortality and (4) health-related outcomes (see ‘Outcomes’ section), using subject index terms and keywords (Table 2). Each database will be searched individually with the search strategy adapted to reflect the differing subject index terms and keywords used by each database. Advanced search features, such as multi-field search, operators, truncation/wildcards and limits, will be combined with the appropriate Boolean terms to create our search strategy. This search strategy will be reviewed by the University of Glasgow College Librarian for Medical, Veterinary and Life Sciences and members of our review panel with expertise in RA and multimorbidity (Online Supplementary File S2).

**Table 2.** Summary of subject index terms and keywords used in search strategy and adapted for each electronic medical database.

Key concepts	Rheumatoid arthritis	Multimorbidity	Mortality	Health-related outcomes
Search index terms and keywords	arthritis, rheumatoid rheumatoid arthritis rheumatic disease*	multimorbid* multi morbid* multiple morbid* multicondition* multi condition* multiple condition* multidisorder* multi disorder* multiple disorder* comorbid* co morbid* condition count*	mortality death surviv* surviv* analys*	function* outcome* function* abilit* function* disability* physical* function* emotion* function* social* function* quality of life chronic pain musculoskeletal pain health assessment questionnaire* patient reported outcome measure* short form questionnaire*

### Study records

#### Data management

The results of the literature search will be downloaded to EndNote (X7.7.1; Thomson Reuters) and duplicates removed. The remaining studies will be uploaded to the systematic review management software, DistillerSR (Evidence Partners, Ontario, Canada), in preparation for the selection process.

#### Selection process

The process for selecting eligible studies will be conducted in two stages, namely title/abstract screening and full-text screening. Titles and abstracts of studies identified from the database searches will be screened independently by the primary researcher (JC) and another member of the review team (SS, BJ, FM or BN) against predefined eligibility criteria. Studies that meet the eligibility criteria will undergo full-text screening. Conflicting studies will also undergo full-text screening. Full-text articles will be obtained and the primary researcher (JC) and one other reviewer (SS, BJ, FM or BN) will again independently review studies against the predefined eligibility criteria. Online supplementary material will be consulted when necessary to support the decision-making process. Disagreements at this stage will be resolved by discussion and/or consultation with a third reviewer. A citation search will also be performed on full-text articles to identify any additional relevant studies.

#### Data collection process

Data from included studies will be extracted and recorded in a predefined data extraction form designed by the primary researcher (JC), as per Cochrane Handbook recommendations, specifically the Cochrane PICO statement where Intervention (‘I’) is replaced with Exposure (‘E’). The form will cover Population, Exposure, Comparator and Outcomes (Table 1). Study characteristics will also be included to record study design, setting, study time period and aims and objectives. The data extraction form will be piloted and any modifications to the form will be made by the review team. Data extraction will be carried out in duplicate by independent reviewers and discrepancies will be resolved by discussion and/or consultation with a third reviewer.

### Data items

#### Study characteristics

Details relating to study design, setting, period of study and study aims and objectives will be extracted.

#### Population

Study population characteristics, such as sample size, sex, age, ethnicity, sociodemographic status, occupation, education, RA duration and treatment, will be extracted. RA definition/diagnosis criteria will be noted. Population recruitment and sampling for each study will be recorded, as will the individual inclusion/exclusion criteria.

#### Exposure

The definition/measure of multimorbidity and number/type of comorbid conditions will be reported for each included study.

#### Comparator

Details of comparator/control groups including definition/measure of participants with RA and no/one other LTCs will be extracted.

#### Outcome

We will record how all-cause mortality and health-related outcomes are defined and measured by each included study. Length of follow-up and statistical analyses used by the authors to evaluate the relationship between RA, multimorbidity and mortality or health-related outcomes will be extracted.

#### Statistical methods

We will also record the statistical methods used, reported results, nature of association reported along with the corresponding effect sizes and adjusting confounders used in the model.

### Outcomes and prioritisation

The primary outcome of interest is all-cause mortality which may be calculated as hazard ratios, odds ratios, incidence rates or survival percentages. Health-related outcomes may be clinician- and/or patient-reported via appropriate questionnaires, for example, HAQ, patient/physician global assessment and short form 36.

### Risk of bias in individual studies

The Quality in Prognostic Studies tool (developed by the Cochrane Prognosis Methods Group) will be used by two reviewers (JC and SS, BJ, FM or BN) to independently assess risk of bias within included studies. Judgements will be made for each of the following domains: Study Participation, Study Attrition, Prognostic Factor Measurement, Outcome Measurement, Study Confounding and Statistical Analysis and Reporting. Each domain will be assigned a ‘Rating of “Risk of bias”’ as either ‘High’, ‘Moderate’ or ‘Low’. The adequacy of reporting will be rated as ‘Yes’, ‘Partial’, ‘No’ or ‘Unsure’. Text comments to facilitate the consensus process will also be included, as necessary. Disagreements between reviewers at this stage will be discussed and resolved by a third reviewer, if required.

### Data synthesis

Included studies will be grouped together according to outcomes in preparation for data synthesis. A narrative synthesis of findings will be conducted which will describe the following from each included study: study characteristics, population, exposure, comparator, outcomes, risk of bias (quality) assessment and study inconsistencies, if any. If appropriate, a meta-analysis will be conducted with tests for publication bias and heterogeneity carried out beforehand. No analysis of subgroups or subsets is planned at present. However, this decision will be re-assessed depending on findings. If appropriate, separate analyses may be conducted on relevant study population characteristics, for example, age, gender, socioeconomic status or multimorbidity count/type of comorbid conditions.

### Ethics approval and dissemination

This systematic review will not contain individual patient data therefore ethics approval is not required. The results of this review will be disseminated via relevant scientific conferences, peer-reviewed publications and social media. This review also fulfils objectives of the primary researcher’s (JC) Doctoral Training Programme (DTP) project.

## Discussion

This systematic review will evaluate the existing literature to determine what is known about the effect of multimorbidity on mortality and adverse health-related outcomes in people with RA. We expect to find multimorbidity to be associated with excess mortality, worsened functional status and reduced quality of life in individuals with RA. We also anticipate that differing numbers and types of comorbid LTCs will have varying impacts on these outcomes. This systematic review will be the first to our knowledge to examine the current information available to delineate such relationships, identify gaps in knowledge and contribute to overall understanding in this area. Identifying areas for additional investigation may further bridge the gap between current understanding and future clinical guideline/health service design modifications.

This systematic review has several key strengths including adhering to PRISMA-P 2015 guidelines to ensure clarity and transparency in our reporting. We will also develop a comprehensive search strategy with guidance from a specialist librarian to minimise the omission of relevant studies. Likewise, all screening and data extraction will be performed independently by two reviewers to limit any oversights.

The definition of multimorbidity and the reporting of outcomes in literature are expected to vary considerably. A narrative synthesis of findings may therefore be conducted to accommodate the heterogeneous nature of the literature which is a potential limitation. The search strategy also restricts publications to English language only which is a possible limitation, although it has previously been suggested that this is not a major drawback to the quality of a systematic review.^11^

Without a defined relationship between RA and multimorbidity, it is difficult to develop and implement standardised treatment advice and guidelines that are inclusive to RA patients with multimorbidity. Enhanced understanding of RA and multimorbidity may therefore help to identify those at increased risk of experiencing adverse clinical and health-related outcomes, with potential implications for future clinical practice.

## Supplemental material

Supplemental Material, Journal_of_Comorbidity_Supplementary_File_S1-v3 - Examining the relationship between rheumatoid arthritis, multimorbidity and adverse health-related outcomes: A systematic review protocol Click here for additional data file. Supplemental Material, Journal_of_Comorbidity_Supplementary_File_S1-v3 for Examining the relationship between rheumatoid arthritis, multimorbidity and adverse health-related outcomes: A systematic review protocol by Jordan Canning, Stefan Siebert, Bhautesh D Jani, Frances S Mair and Barbara I Nicholl in Journal of Comorbidity

Supplemental Material, Journal_of_Comorbidity_Supplementary_File_S2-v3 - Examining the relationship between rheumatoid arthritis, multimorbidity and adverse health-related outcomes: A systematic review protocol Click here for additional data file. Supplemental Material, Journal_of_Comorbidity_Supplementary_File_S2-v3 for Examining the relationship between rheumatoid arthritis, multimorbidity and adverse health-related outcomes: A systematic review protocol by Jordan Canning, Stefan Siebert, Bhautesh D Jani, Frances S Mair and Barbara I Nicholl in Journal of Comorbidity
