# Supplementary material for: Examining the relationship between rheumatoid arthritis, multimorbidity and adverse health-related outcomes: A systematic review protocol
Source: J Comorb. 2020 Mar 16;10:2235042X20906657. doi: 10.1177/2235042X20906657 (PMC7079302; doi:10.1177/2235042X20906657)
Supplement: Supplemental Material, Journal_of_Comorbidity_Supplementary_File_S2-v3 - Examining the relationship between rheumatoid arthritis, multimorbidity and adverse health-related outcomes: A systematic review protocol [file Journal_of_Comorbidity_Supplementary_File_S2-v3.pdf]

Supplementary File S2: Search strategy draft to be used in MEDLINE.

Search No.	Subject index terms and keywords
1	rheumatic diseases/ or arthritis, rheumatoid/
2	("rheumatic disease*" or "rheumatoid arthritis").tw.
3	1 or 2
4	comorbidity/ or multimorbidity/
5	("multimorbid*" or "multi morbid*" or "multiple morbid*").tw.
6	("multicondition*" or "multi condition*" or "multiple condition*").tw.
7	("multidisease*" or "multi disease*" or "multiple disease*").tw.
8	("multidisorder*" or "multi disorder*" or "multiple disorder*").tw.
9	("comorbid*" or "co morbid*").tw.
10	("comorbid* count*" or "co morbid* count*" or "condition count*").tw.
11	4 or 5 or 6 or 7 or 8 or 9 or 10
12	Mortality/
13	("mortality" or "death" or "surviv*" or "surviv* analys*").tw.
14	12 or 13
15	chronic pain/ or musculoskeletal pain/
16	("musculoskeletal pain" or "chronic pain").tw.
17	("function* outcome*" or "function* abilit*" or "function* disabilit*").tw.
18	("physical* function*" or "emotion* function*" or "social* function*").tw.
19	mental health/ or anxiety/ or depression/
20	("mental health" or "anxiety" or "depression").tw.
21	"Quality of Life"/ or Sickness Impact Profile/
22	("quality of life" or "life quality" or "health-related quality of life" or "health related quality of life" or "life experience*" or "sickness impact profile").tw.
23	Patient Reported Outcome Measures/
24	("patient reported outcome measures" or "PROMs").tw.
25	("health assessment questionnaire*" or "health assessment questionnaire disability index" or "HAQ*" or "short form questionnaire*" or "short form surv*").tw.
26	15 or 16 or 17 or 18 or 19 or 20 or 21 or 22 or 23 or 24 or 25
27	exp Adult/
28	("adult" or "aged" or "middle aged" or "young adult" or "aged, 80 and over" or "elderly").tw.
29	Humans/
30	("human" or "humans").tw.
31	27 or 28 or 29 or 30
32	14 or 26
33	3 and 11 and 31 and 32
34	limit 33 to english
35	3 and 11 and 32
36	limit 35 to (english language and humans and "all adult (19 plus years)")
